# The Cytokine Network of Acute HIV Infection: A Promising Target for Vaccines and Therapy to Reduce Viral Set-Point?

**DOI:** 10.1371/journal.ppat.1002055

**Published:** 2011-08-11

**Authors:** Peter D. Katsikis, Yvonne M. Mueller, François Villinger

**Affiliations:** 1 Department of Microbiology and Immunology, Drexel University College of Medicine, Philadelphia, Pennsylvania, United States of America; 2 Department of Pathology and Laboratory Medicine, Yerkes National Primate Research Center, Emory University, Atlanta, Georgia, United States of America; University of California San Diego, United States of America

## Abstract

Cytokines play a central role in the pathogenesis of many diseases, including HIV infection. However, the role of the cytokine network in early HIV infection is only now starting to be elucidated. A number of studies conducted in recent years have indicated that cytokines of the acute/early stages of HIV and SIV infection can impact viral set-point months later, and this is of critical importance since viral set-point during chronic HIV infection affects virus transmission and disease progression. This raises the question whether modulating the cytokine environment during acute/early HIV infection can be a target for novel approaches to develop a vaccine and therapeutics. In this review we focus on the kinetics and function of cytokines during acute HIV and SIV infection and how these may impact viral set-point. We also discuss unresolved questions that are essential for our understanding of the role of acute infection cytokines in HIV infection and that, if answered, may suggest novel therapeutic and vaccine strategies to control the worldwide HIV pandemic.

## Introduction

Early events during acute HIV infection may determine progression and pathogenesis of infection, as the immunological milieu of the initial antigen encounter appears critical in dictating the long-term equilibrium between the host and the pathogen [1]. This early period, which includes the eclipse phase before viremia is detected and the viremic phase before viral set-point is reached, is critical for target cell availability, seeding of latent reservoirs, and the initiation and expansion of antiviral immune responses by the host. While such events have been difficult to assess in humans [2], [3], animal models such as rodent and the non-human primate model of AIDS have afforded us the opportunity to address such seminal questions. Thus, chronic immune stimulation [4], [5], immunosuppression [6], partial virus-specific immunity [7], and/or the use of cytokines [8]–[11] or inhibitors of cell death [12] have all been shown to alter not only the viral replication dynamics and quality of immune responses, but more importantly also the kinetics of disease progression. Among these immunomodulatory approaches, cytokines provide one of the most targeted factors to investigate alterations of the viral kinetics, the recruitment of viral targets, and the development of anti-viral immunity.

## Cytokine Milieu in Early/Acute HIV/SIV Infection

The complexity of the role of the cytokine milieu in acute HIV and SIV infection has only partially been addressed. The first reports examining cytokines in acute HIV infection were conducted in patients with symptomatic acute infection [13], [14]. However, very early events during the first days and weeks could not be assessed since the exact time of infection was unknown and the symptomatic phase can occur several weeks after initial viral exposure [3]. A more recent study analyzed plasma cytokines in HIV infection after the eclipse phase in patients with detectable viral load (at least 100 HIV RNA copies/ml) [15]. This examination of systemic plasma cytokines revealed that IFNα and IL-15 were the first cytokines elevated within 5 days after detection of viremia, followed by TNFα, CXCL10, and IFNγ, and then by IL-12 [15]. As expected for the anti-inflammatory cytokine IL-10, increased IL-10 mRNA and protein levels are detected rather late in HIV infection, after the increased expression of proinflammatory cytokines [15], [16]. Another well-known inhibitory cytokine upregulated in the majority of acutely HIV infected individuals is IL-1R antagonist (IL-1Rα) [15]. In vitro, IL-1Rα inhibits IL-1-mediated HIV replication [17], suggesting that IL-1Rα would suppress viral replication during acute infection. Similar to IL-10, however, IL-1Rα may also affect anti-viral immunity. A major caveat in all of these human studies is the estimated time point of infection.

**Figure 1:**
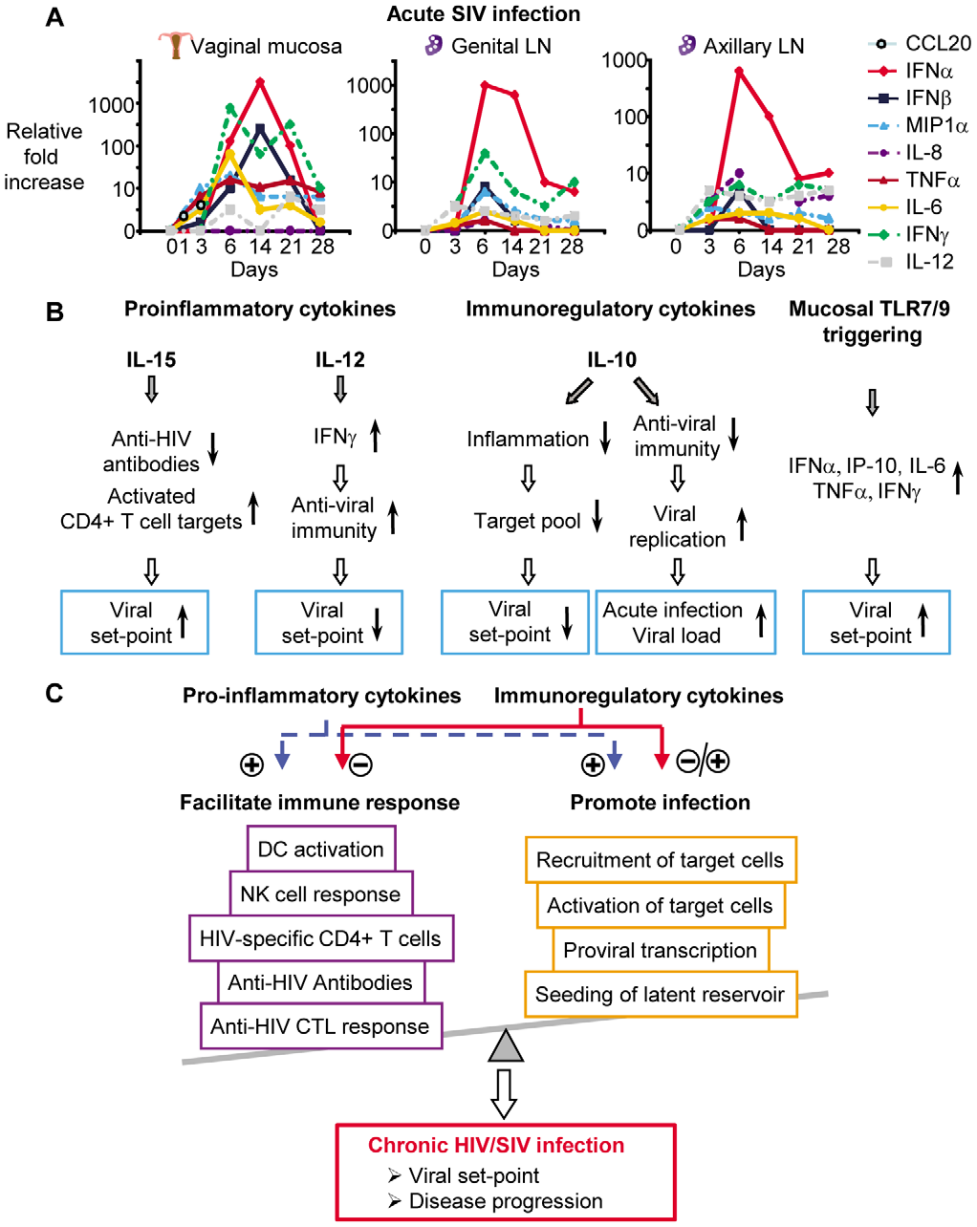
Early cytokines determine pathogenesis of HIV/SIV infection. (A) Several cytokines are produced during acute SIV infection, either at the site of initial viral exposure or in draining or distal lymphoid tissue (cytokine mRNA kinetics are adapted from [18], [22]; scale depicts fold changes over time for each individual cytokine). (B) Cytokines either increase (↑) or decrease (↓) immune parameters or target availability and therefore directly influence viral replication and viral set-point (effects of cytokines are adapted from [8], [10], [23], [66], [70]). (C) Pro-inflammatory and immunoregulatory cytokines either negatively or positively modulate immune responses and viral replication and this determines viral set-points and disease progression during chronic HIV/SIV infection.

A more precise timing of cytokine kinetics, however, can be done in SIV-infected non-human primates. Several such studies have been conducted of very early SIV infection in non-human primates to analyze the cytokine production during the first weeks of infection and compare differences between non-pathogenic and pathogenic infections [2], [18]–[25]. Some of the first studies examined cytokine mRNA after intravenous (i.v.) SIV infection in tissues as the viral dynamics evolved. By day 7 post i.v. infection, IL-10 mRNA was detected in bronchial lavage cells but not lymph nodes (LNs) or peripheral blood mononuclear cells while IFNγ mRNA was detected later [26], [27]. Another study, however, indicated that IFNγ mRNA is upregulated in LNs at day 7 while IL-2 and IL-12 mRNA increase after day 14 [28]. When plasma cytokines were measured, IL-12 and IL-18 were found to be induced after 2 weeks of infection, whereas IFNα/β was detected already by week 1 [29]. While these studies provided seminal observations, the i.v. route of infection used does not mirror the predominant route of infection in humans where virus infection and cytokine production start at mucosal tissues and spread distally. Within 24 hours following mucosal infection, endocervical epithelium produces MIP-3α (CCL20) [22], a chemokine involved in recruitment of plasmacytoid dendritic cells (pDCs). Such subepithelial pDCs are recruited and produce IFNα, IFNβ, MIP-1α (CCL3), and MIP-1β (CCL4) at day 1 of infection, which in turn promote inflammation and recruit T cell targets for infection [22]. Subsequently, IL-8 and RANTES are detected in mucosal sites and these further enhance local inflammation [22]. After the initial mucosal infection, the virus spreads within days into the local draining LNs and then subsequently to more distal LNs [30], [31]. When cytokine mRNA expression was evaluated in vaginal mucosal tissue, genital tract draining LNs, and distal LNs, MIP-1α, TNFα, and IL-6 were detected at days 3–5 in all tissues. IFNβ was only detected in vaginal mucosa at these early time points [18] (Figure 1A). IFNα was observed in all tissues at days 6–10, whereas IL-12 and IFNγ were detected in draining and distal LNs at days 3–5 and later at days 6–10 in the vaginal mucosa. Cytokine mRNA declined in parallel with the decrease of viral replication after day 14, suggesting that the virus directly or indirectly drives much of the cytokine production [18]. Similar to the human studies above, IL-15 is detected systemically in plasma during acute SIV infection within a few days of i.v. infection and peaks at day 10 [32]. Limited data is available for kinetics of immunosuppressive cytokines during acute SIV infection. Such cytokines can act as a double-edged sword, as they can dampen virus-specific immunity but also inhibit infection by reducing T cell recruitment and activation. Immunosuppressive cytokines such as IL-10 and transforming growth factor-β1 (TGFβ1) are also present during acute SIV infection. In SIV-infected rhesus macaques, IL-10 production in LNs is already detected at day 7 and increases further by day 28 post-infection [33]. Increased TGFβ1 in LNs is also observed early in SIV-infected rhesus macaques by day 7, with production peaking on day 12 [33].

Comparison of cytokine gene expression profiles and kinetics in the non-pathogenic SIV infection of natural hosts (African green monkeys and sooty mangabeys) with pathogenic SIV infection of non-natural hosts (rhesus macaques and pigtailed macaques) can be used to link cytokine production with pathogenesis. IFNα, TGFβ, and IL-10 mRNA and protein expression is increased earlier in non-pathogenic SIV infection compared to pathogenic SIV infection [20], [21], [34], and this is accompanied by transient IFNγ expression only in non-pathogenic SIV infection [20]. These data suggested that although natural hosts of SIV upregulate cytokines like IFNα and IFNγ, these cytokines are downregulated much faster. This is paralleled by a much earlier production of regulatory cytokines, indicating an important role of these cytokines in controlling SIV pathogenesis. Comparison of non-pathogenic and pathogenic SIV infection has suggested a possible role in HIV/SIV pathogenesis for IL-17. IL-17 is produced by several cell types, including Th17 cells, natural killer T (NKT) cells, and γδ T cells (reviewed in [35]). Th17 cells are depleted during acute SIV infection from peripheral blood in pathogenic but not in non-pathogenic SIV infection [36]. During chronic infection, Th17 cells are significantly reduced in pathogenic SIV infection in lymphoid tissue [36] and gastrointestinal track [37], [38], which likely impairs control of intestinal flora and pathogens [37]. In contrast, increased numbers of IL-17-producing NKT cells are detected at day 14 in LNs of pathogenic but not non-pathogenic SIV infection [39]. Since IL-17 overproduction can promote inflammation [40], increased IL-17 secretion in LNs during acute HIV/SIV infection could lead to increased recruitment of target cells, viral dissemination, and malabsorption. Clearly, IL-17 may have an important impact on the pathogenesis of infection and further studies are required to address this. Although comparisons of non-pathogenic and pathogenic SIV infection may reveal important aspects of how cytokines contribute to pathogenesis, they are of limited use for studying the role of cytokines on viral set-point as non-pathogenic SIV infection of natural hosts is accompanied by high viral loads.

## Source of Cytokines during Acute HIV/SIV Infection

The source and location of cytokine production during acute HIV/SIV infection is only partially understood. Cytokines in the very early phase of HIV and SIV infection are most likely produced locally, whereas after virus dissemination, cytokines are produced by more generalized and distant sites such as genital draining and distal LNs and gut-associated lymphoid tissue [30]. The human studies have relied on measuring cytokines in plasma [15], and their kinetics suggest they reflect systemically produced cytokines after dissemination beyond the site of entry. In SIV infection, several cell types present in the vaginal mucosa and LNs have been shown previously to produce cytokines during acute infection. Type I IFN is primarily produced by plasmacytoid DCs [41], IL-12 by conventional DCs and macrophages [42], [43], and IL-15 by many different cell types, including DCs and macrophages [44]–[46]. As mentioned above, the epithelium at the local port of entry produces MIP-3α (CCL20), and recruits/activates pDCs to produce IFNα, IFNβ, MIP-1α (CCL3), and MIP-1β (CCL4) [22]. Once the virus spreads to the draining LNs, T cells, monocytes, and pDCs produce IFNα, IL-12, IFNγ, IL-10, and TGFβ1 in draining and distal LNs [18], [33]. Despite the recent progress at dissecting cytokine production during acute infection, much more is needed to identify the cell types and respective location of cytokine production and how these relate to plasma levels during the disseminating acute infection. This latter point is critical for our understanding of the human studies that cannot easily access tissues and rely on plasma measurements.

## Viral Set-Point and Cytokines during Acute HIV/SIV Infection

A central question regarding cytokines during acute HIV/SIV infection is whether these cytokines are beneficial or detrimental for the host. Cytokines can upregulate or accelerate the anti-viral immune response, and this could contribute to viral control [47] . However, at the same time, these cytokines can also increase the target cell pool for HIV/SIV by recruiting and activating CD4+ T cells, the primary targets for HIV/SIV infection. Immunosuppressive cytokines can dampen anti-viral immunity but also reduce inflammation and decrease the target cell pool of activated CD4+ T cells. Most cytokines exert pleiotropic and sometimes contrasting effects on the immune response and viral replication as suggested by a number of studies. Examples of these are pro-inflammatory cytokines such as TNFα that are produced during acute HIV/SIV infection [15], [18] and increase anti-viral immunity but also induce NF-κB [48], which enhances proviral transcription and drives HIV replication [49]–[51]. IL-15 can enhance NK cell and CD8+ T cell responses in HIV and SIV [52]–[54] but also correlates with increased viral replication [32].

Recent studies have suggested that cytokines during acute HIV/SIV infection are important determinants of the viral set-point of chronic infection [8], [10], [15], [39], [42], [55]. Viral set-points are stable in anti-retroviral naïve, asymptomatic, chronically HIV-infected patients but can vary greatly between patients. In patients on highly active antiretroviral therapy (HAART) for a prolonged time, viral set-points seen after discontinuation of HAART match pre-treatment set-points [56]–[58]. Experimental depletion of CD4+ T cells in monkeys only temporarily lowered the set-point [59], indicating a remarkable individual stability. Viral set-points correlate with disease progression with higher viral set-points being associated with a more rapid progression of HIV [60], [61]. Reduced viral set-point during chronic HIV infection is associated with reduced heterosexual and maternal–infant transmission of HIV [60]–[65]. Untreated HIV-infected women with a viral set-point below 1,500 HIV-1 RNA copies/ml plasma fail to sexually transmit the virus [64]. The risk of maternal–infant HIV-1 transmission is reduced in pregnant HIV-infected individuals with low viral set-point [62], [63], [65]. Therefore, understanding how cytokines of acute HIV infection can affect viral set-points may suggest novel approaches to decrease this important determinant of disease progression and transmission.

An association of acute infection cytokines with viral set-point was recently suggested in HIV infection. Higher plasma IL-7 and IL-15 levels during acute/early HIV infection appear detrimental and correlate with higher viral set-points, while conversely, plasma IL-12 and IFNγ levels appear beneficial and associate with lower viral set-points [55]. A protective role of early IL-10 upregulation was also indicated in studies, which showed that individuals with polymorphisms resulting in increased IL-10 production have lower viral set-points [23], [66]. These human studies have suggested that cytokine profiles during acute infection may determine viral set-point, and non-human primate studies have provided further support for this.

One prediction from the above human studies would be that addition of cytokines during acute SIV infection would alter viral set-point months later. Such studies have been conducted and remarkably agree with these predictions based on plasma level correlations in HIV infection. Treatment of acute SIV infection with simian IL-15 resulted in a dramatic 1,000-fold increase in viral set-point [10], [67] (Figure 1B). IL-15 treatment of acute SIV infection accelerated disease progression [10] while treatment during chronic infections did not affect viral set-points or disease progression [52]. In contrast, when IL-12 was administered to rhesus macaques acutely infected with SIV, it lowered viral set-point by 100-fold [8] (Figure 1B) and inhibited disease progression [8]. Treatment of chronic SIV-infection with IL-12 did not affect viral set-point or disease progression [68], [69].

That cytokines present early during acute infection can affect viral set-point is further supported by a study testing whether the induction of antiviral cytokines at the vaginal mucosa would prevent SIV infection. The basic premise for this study was that Toll-like receptor (TLR) 7 and 9 agonists applied intravaginally would stimulate antiviral cytokines and induce an antiviral state in the vaginal mucosa. As expected, these agonists upregulated mRNA and protein for several cytokines in vaginal secretions, including IFNα, TNFα, IL-6, IP-10, and IFNγ [70]. Despite the induction of such cytokines, animals treated with TLR agonists were readily infected, exhibiting increased viral set-points compared to untreated animals [70] (Figure 1B). Peak viremia post-infection, however, was not affected, and this is similar to IL-15 and IL-12 administration during acute infection [8], [10], [67].

The potential mechanisms by which acute HIV/SIV infection cytokines affect viral set-point are suggested by the above studies. Although the precise mechanism by which IL-15 is affecting viral set-point is not known, effects on host immunity and/or infection of target cells are suspected, as IL-15 decreases anti-SIV antibody responses but enhances the homeostatic proliferation of predominantly memory CD4+ T cells during acute infection, and both correlate with increased viral set-point [10]. IL-15’s effect on viral set-point occurred despite an increase in virus-specific CD8+ T cell and NK cell numbers in these animals [10]. The IL-15-mediated increase in CD4+ T cell proliferation may accelerate or enhance the loss of SIV-specific CD4+ T cells and this would explain the reduced anti-SIV antibody levels. On the other hand, increased CD4+ T cell proliferation could augment the seeding of infected cells. Of interest, IL-12 treatment during acute infection markedly promoted the recruitment and activation of NK cells similar to IL-15. IL-12 however, also induced a rapid production of IFNγ in these animals [8], and/or prevention of apoptosis of SIV-specific CD4+ T cells [71], which prevented contraction of effector T cells and enhanced anti-viral immunity [12], [72]. The preservation of newly minted antiviral CD4+ and CD8+ T cells may be key to downstream control of viremia and disease progression [6], [8], [12], [72]. The intravaginal application of TLR agonists induced the host to produce cytokines that have both anti-viral and pro-inflammatory activity, including IFNγ [70]. This cytokine induction was accompanied by the markedly enhanced recruitment of activated CD4+ T cells, macrophages, and DCs in animals treated with TLR agonists fueling the initial foyer of infection [70]. These seemingly contradicting results with overlapping profiles of cytokines highlight our lack of understanding about cytokine profiles and kinetics that are ultimately beneficial versus those that are deleterious to the newly infected host during acute HIV/SIV infection

## Can Knowledge of the Cytokines Network in Acute HIV/SIV Infection Enable Us to Design New Therapeutic and/or Vaccine Strategies?

From the data discussed above, it is clear that without a better understanding of the complex interactions between cytokines and the host during acute HIV/SIV infection, the accurate prediction of the effect of cytokine interventions will be difficult (Figure 1C). A number of critical questions remain unanswered. Thus, what are the precise cytokine profiles of acute infection as the infection spreads from mucosal sites into lymphoid tissue? From what cell types and tissues are these cytokines being made during acute infection? What is the source of systemically detected cytokines? What triggers their production? Are some cytokines induced by pattern recognition receptors directly triggered by virus and others by virus-specific immunity? What is the window of opportunity during acute HIV/SIV infection for cytokine manipulations to affect viral set-point? How are acute infection cytokines affecting viral set-point? Are cytokines affecting viral set-point by altering innate and adaptive immunity, availability and infectivity of target cells, or evolution and fitness of the virus?

Some of these questions can now be answered, as assays to measure multiple cytokines in non-human primates are being developed and validated. To investigate the cytokine profile in plasma and tissues, the cell type source and the production location will require a concerted effort of multiple laboratories specializing in non-human primate studies. Importantly, understanding the role and function of individual cytokines in determining viral set-point will require the inhibition or addition of individual cytokines during acute SIV infection. These types of experiments require significant resources for the production and validation of cytokines and blocking reagents. Finally, both extensive immunological and virological studies need to be conducted in the setting of cytokine administration or inhibition during acute SIV infection to determine the mechanism by which viral set-points are altered.

Findings from studies above can help identify cytokines that can be targeted to alter HIV viral set-point, transmission, and disease progression. This could lead to cytokines or cytokine inhibitors being used as pharmacological agents in acute/early infection with the goal of altering the course of infection and lowering viral set-points. Cytokine hierarchies have been exploited therapeutically in patients with rheumatoid arthritis (RA) and inflammatory bowel disease (IBD) where blocking TNFα, a key driver of the inflammatory cytokine network in RA and IBD, has a major impact on inflammation [73]–[75]. Blocking cytokines is clearly feasible but its applicability in HIV infection will depend on the window of opportunity after initial exposure that still enables the reduction of the viral set-point. Another strategy would be to develop vaccines that imprint the cytokine network. Such vaccines would be non-sterilizing as the host would acquire infection but could potentially lower viral set-points below critical levels required for HIV transmission. In addition, such vaccines could be combined with vaccines designed to prevent acquisition at the mucosal surface [76], thereby providing a double barrier. Adjuvants can dictate the development of Th1 or Th2 immunity [77]. Toll-like receptor ligands, for example, can direct the cytokine profile of immunity against pathogens [78]. When *Leishmania* and *Mycobacterium tuberculosis* are formulated in oil-in-water emulsions they primarily induce Th2 responses, whereas the addition of TLR4 agonists such as monophosphoryl lipid A or glycopyranosyl lipid [79], [80] directed their response to a Th1 profile [78]. Several recent studies utilized cytokines as vaccine adjuvants with some promising results, such as IL-15-leading to increased levels of CD8+ T cell responses [81]–[83]. Such adjuvant or cytokine approaches could be used to develop vaccines which imprint an immune response to HIV that results in a beneficial cytokine profile or network during acute HIV infection of the host.

## Conclusion

We have reviewed above the evidence that viral set-point can be manipulated by cytokine interventions during acute HIV/SIV infection. This raises the question whether we can target cytokines during acute HIV infection to alter viral set-point through immunotherapeutics and potentially vaccines. Treating with cytokines or blocking cytokines is clearly feasible but will depend on the window of opportunity after initial HIV exposure, during which such treatments are still effective at lowering viral set-point. Non-sterilizing vaccine strategies that alter the cytokines produced during acute infection will be challenging, but the reward of a vaccine that reduces viral set-point below transmission levels would be tremendous. Further understanding of the cytokine network and the potential cytokine hierarchies during acute HIV/SIV infection may uncover critical cytokines that control viral set-point. This may not only provide fundamental insight into the poorly understood mechanisms that control viral set-point but could instruct the development of novel immunotherapeutics or vaccine adjuvants against HIV.
